# Eligibility to donate blood under an individual donor assessment policy: A national cross-sectional study of gay, bisexual and other men who have sex with men in Aotearoa, New Zealand

**DOI:** 10.1371/journal.pone.0354717

**Published:** 2026-07-23

**Authors:** Peter J. Saxton, Celesti Tan, Sarah Morley, Patricia Priest, Susan M. McAllister, Stephen Ritchie, Janine Paynter, Kevin Haunui, Cameron Leakey, Joseph Rich, Mark Fisher, Koson Tony Sriamporn

**Affiliations:** 1 Faculty of Medical and Health Sciences, University of Auckland, Auckland, New Zealand; 2 New Zealand Blood Service, Auckland, New Zealand; 3 Department of Public Health Dunedin, University of Otago, Dunedin, New Zealand; 4 Burnett Foundation Aotearoa, Auckland, New Zealand; 5 Body Positive Inc, Auckland, New Zealand; University of Technology Sydney, AUSTRALIA

## Abstract

**Background:**

To protect the blood supply, many countries defer higher-risk donors such as men who have had sex with men (MSM) in the last 3 months. However, this may be unnecessarily restrictive and increase non-compliance risks. New Zealand (NZ) has shifted from this approach to individual donor assessment (IDA), but the potential impact is poorly understood. We aimed to estimate the increase in blood donor eligibility and examine the characteristics of newly eligible MSM.

**Methods:**

We used data from SPOTS, a large national cross-sectional online survey of MSM. Items included socio-demographics, blood donation history, views and preferences about blood donation, sexual behaviours and drug use. We described blood donation engagement and estimated the proportion eligible to donate blood under three scenarios (previous NZ policy; UK-style: IDA with chemsex deferral; Canada-style; IDA without chemsex deferral). We then examined the characteristics of potentially newly eligible MSM donors.

**Results:**

Of the 3,235 participants, 43.1% had ever donated blood, 82.0% were interested in donating, 86.1% preferred an IDA-style policy, and 80.6% intended to donate, should they become eligible. We estimated 13% were eligible to donate under the previous NZ policy, rising to 37% under a UK-style IDA, and 41% under NZ’s new IDA policy. In NZ’s new IDA policy, 30% of currently ineligible participants will become eligible. Compared to participants currently eligible, newly eligible MSM were more likely to be aged 30–44 (OR 1.8, 1.4–2.4), tertiary educated (OR 1.4, 1.1–1.8), spend a lot of their free time with gay men (OR 4.3, 3.2–5.9), and identify as gay only (OR 2.5, 1.96–3.2). As expected, a high proportion of newly eligible MSM were in a regular same-sex relationship.

**Conclusions:**

NZ could triple eligible MSM donors with its new IDA policy. Findings from this research are relevant internationally as blood services plan IDA implementation.

## Background

A safe and self-sufficient blood supply is crucial for medical care. To achieve this, countries employ a combined approach of testing all blood for known pathogens, methods to remove or destroy pathogens that might be present in a donation, and deferring individuals with a higher probability of a recently-acquired undiagnosed transfusion-transmissible infection, such as HIV [1]. Deferral of some groups remains necessary because even state-of-the-art testing can miss infections in the early ‘window period’, placing blood recipients at risk [2]. At the same time, overly strict deferral policies can jeopardise the blood supply and should be regularly reviewed [2,3]. In recent years, blood services internationally have amended the deferral criteria for men who have sex with men (MSM), in response to advances in screening and processing blood products, and claims that policies were discriminatory, unscientific, and dissonant with contemporary HIV prevention advice [4]. What is less clear are the implications of deferral policy liberalisation on the expected number and characteristics of newly eligible MSM blood donors.

Reforms to blood donor deferral policies in Aotearoa New Zealand (NZ) have closely followed changes in the United Kingdom (UK), Canada, and Australia. The NZ Blood Service (NZBS) introduced a ten-year deferral policy in 1998 for men having oral or anal intercourse with a man (with or without a condom), which was shortened to five years in 2008, 12 months in 2014 and three months in 2020 [2,3,5]. Such deferrals have been justified on the basis that MSM are the population most affected by HIV in NZ, historically accounting for over 75% of local HIV transmission, having a relatively high prevalence of undiagnosed HIV (1.3% in 2011), and being approximately 350 times more likely to be diagnosed with HIV than heterosexual New Zealanders [6–8]. However, the last decade has seen dramatic improvements in HIV prevention. The annual number of new locally acquired HIV diagnoses among MSM decreased 61% between 2016 and 2024, and uptake of effective HIV prevention practices and HIV testing has increased [9,10]. NZ’s record on blood safety is also excellent. The prevalence of major blood-borne viruses in NZ donors is low and has not changed significantly since 2008 [2]. Additionally, there have been no reported cases of HIV transmission by tested blood in NZ since antibody testing was introduced in 1985, with the current risk of HIV transmission via blood transfusion estimated at 1 in 9.6 million donations [2].

Many MSM view giving blood as a way of contributing to the common good that is unnecessarily being denied to them (and to the blood recipients they could help) [11]. Consequently, the continued deferral of blood donations from most MSM, despite their generally low absolute risk of HIV, has become controversial. In NZ, MSM in monogamous relationships have expressed resentment at blood services for their ineligibility and MSM engaging in safe sex perceive a double standard [11]. Restrictive deferral policies, therefore, not only limit the pool of available donors but potentially reduce blood safety if frustration and negative perceptions lead to non-compliance with deferral rules [12]. Broadening donor eligibility criteria could mitigate such risks, depending on who and how many MSM could donate.

In line with other countries, the NZBS announced a change in donor deferral policy, shifting from blanket time-based deferrals for MSM to a gender-neutral individual donor assessment (IDA) [13]. This change noted the consistent evidence of no increase in HIV or other transfusion-transmissible infections following the implementation of more inclusive, individualised criteria in other jurisdictions [4,14–16]. For example, the UK implemented a gender-neutral IDA policy in 2021 and has reported no deterioration in the safety profile of donated blood [17,18]. Canada, whose gender-neutral IDA policy implemented in 2022 is slightly less stringent than the UK (“chemsex” is not a grounds for deferral of MSM in Canada), was also a possible model for NZ [4,19]. The implementation of the IDA policy in Canada has similarly led to no difference in the detection of HIV in donated blood [20]. For NZ, these international findings demonstrate how IDA has been implemented in comparable settings. In May 2026, the NZBS formally introduced NZ’s new IDA policy, that was based on Canada’s IDA policy. Local data are now needed to understand MSM donation patterns to estimate their previous and prospective eligibility.

Using a large and diverse online sample of MSM recruited in NZ, the aim of this study was to describe the engagement of MSM in blood donation and future policy preferences, estimate the potential eligibility of MSM in our sample to donate blood under different scenarios (previous NZ policy, UK-style policy, new NZ policy) and examine the socio-demographic characteristics and HIV-risk reduction behaviours of MSM who are likely newly eligible under NZ’s new IDA policy.

## Methods

### Study design and recruitment

The Sex and Prevention of Transmission Study (SPOTS) is a national cross-sectional behavioural surveillance survey about sex between men, HIV prevention, and blood donation in NZ. Participation was voluntary, anonymous, and self-completed online. Participants were recruited using advertisements on social media, gay dating mobile applications, pornography websites, national television and print media, community mailing lists, and posters in public and gay-friendly venues across NZ between 26 April and 4 August 2022. People were eligible if they were aged 16 years or over, lived in NZ, and were a man (cis or trans) who had ever had sex with a man, or identified as gay, bisexual or non-heterosexual, or were a trans woman or non-binary person who had had sex with MSM in the previous five years. Written informed consent was obtained prior to the online questionnaire hosted on Salesforce (San Francisco, CA, USA)/SurveyTitan. As no participants were minors, parental or guardian consent was not required. The study was funded by the Health Research Council of New Zealand (Ref 20/887) and was approved by the New Zealand Health and Disability Ethics Committee (HDEC 2021 EXP 11450). A full description of the behavioural surveillance methods have been reported elsewhere [21,22].

### Measures

Socio-demographic items included age, ethnicity, highest education level, and sexual identity. We categorised age into three groups (under 30 years, 30–44, 45 years or more). Participants were able to identify with multiple ethnic groups and we dichotomised participants into those reporting ‘European only’ versus those reporting any other ethnicity. Responses to highest education level (less than tertiary/tertiary or more) and sexual identity (gay identified only versus bisexual, pansexual, queer, takatāpui or another identity) were also dichotomised.

Behavioural items included questions on free time with gay men (a lot versus some, a little or none), current relationship status with a man (monogamous, non-monogamous or unsure, no current regular male partner) and anal intercourse with a man within the last 6 months (yes, no). We categorised HIV testing status into four groups (tested negative within the last 6 months, tested negative more than 6 months ago, never HIV tested, diagnosed HIV positive). PrEP status classified participants into those who had not taken PrEP in the last 6 months, those who had used PrEP in the last 6 months, and those who were ineligible (HIV positive).

Blood donation items included blood donation history (ever donated blood, never donated). Participants who had ever donated blood were presented with options about why they had, with multiple responses allowed (e.g., “I believed I was low risk for HIV”, “I was compliant with the policy at the time”, “I had not recently had oral or anal sex with a man”). Those who had never donated blood were asked why they had not (e.g., “I’m not interested”, “I was deferred (I tried to donate but was asked not to at the point of donation)”. We gauged interest in donating blood (yes, no), awareness of NZ’s blood donation rules at the time of survey (aware, not aware), about the blanket MSM deferral (“If you’re a man you are asked not to donate blood for 3 months following anal or oral sex with a man, with or without a condom”), about deferral related to PrEP use (“People are asked not to donate blood for 3 months following their last pre-exposure prophylaxis medication”) and about deferral of people living with HIV (“People living with HIV are never allowed to donate blood, even if they are taking HIV antiretroviral medications and have an undetectable viral load”). Participants were asked what their future policy preference would be, with two options: “I support the current policy, for example, being asked a simple broad question (e.g., when did you last have sex with a man), but being asked not to donate if I have had sex within a certain timeframe”, or “I support a more tailored policy, for example, more detailed personal questions about my behaviour, if it potentially allowed me to donate sooner”. A measure of a participant’s intention to donate blood was asked using one question: “If I became eligible in the future, I intend to donate blood” using a seven-point Likert scale ([1] “strongly disagree” to [7] “strongly agree”). Responses were dichotomised into those not intending to donate blood [1–3] or neutral [4] versus those intending to donate [5–7].

### Analysis

For all analyses, we limited the sample to participants who reached the blood donation section of the questionnaire. We also excluded trans women and non-binary people assigned female at birth, as the current blood donor policy concerns men who have sex with men.

We determined the prevalence of key findings regarding blood donation engagement (donation history, awareness, interest, preferences and intentions). We calculated the potential blood donation eligibility status of participants based on three scenarios: (1) NZ’s previous 3-month blanket MSM deferral policy set in 2020 that ended in April 2026, (2) a hypothetical scenario based on the UK IDA policy, and (3) a hypothetical scenario based on the Canadian IDA policy. To estimate these, we drew on available SPOTS questions relevant to the deferral criteria in each setting. The research team based these decisions on findings from published literature complemented by advice from an international expert advisory group with representatives from NZ, UK, Canada, Australia and the United States (USA). These included items on age when presenting for donation, HIV status (of participant and partner), hepatitis C virus (HCV) status, sexually transmitted infection (STI) history including syphilis and gonorrhoea, PrEP, timing of last sex with a man, anal intercourse, injecting drug use (IDU), chemsex, and sex work (Table 3). Because the SPOTS items did not always exactly match the deferral criteria in each setting, we operationalised them for the purposes of these estimates (see footnotes in Table 3). For these estimations and all subsequent analyses, we further limited the sample to those reaching the end of the questionnaire (to reduce missing data) and to those who provided a response to the timing of last sex they had with a man, a key criterion for all deferral settings.

Using our donor eligibility estimates, we described the potential future MSM population who will become eligible to donate blood in NZ, to inform planning by blood donation services. For this, we assumed NZ would adopt a Canada-style IDA policy, which the NZ Blood Service ultimately did in May 2026. We identified three groups, those who were: (1) not previously eligible to donate in NZ nor likely to be in a new NZ IDA policy based on Canada’s, (2) previously eligible to donate blood in NZ under the 3-month blanket MSM deferral, (3) not previously eligible to donate but becoming eligible under a new NZ IDA policy based on Canada’s. We described the socio-demographic characteristics and behavioural profile of each group. We then compared the socio-demographic characteristics of those previously eligible versus those who will be eligible in the new NZ IDA policy (i.e., the “newly eligible population” from May 2026). We examined this using crude odds ratios (OR) with 95% confidence intervals (CI) from a logistic regression with future eligibility set as the dependent variable.

## Results

Of the 3,838 SPOTS participants, 3235 (84%) reached the blood donation section and were eligible for analysis (Table 1). Two-fifths were aged under 30 years, more than a quarter (27.8%) identified with at least one non-European ethnicity, over half (56.4%) had a tertiary education, and 36.3% claimed at least one sexual identity that was different to “gay” (although they may have claimed gay as well). Two thirds (66.4%) spent “a lot” of their spare time with other gay and bisexual men, around half reported being either in a current monogamous (23.4%) or non-monogamous (27.6%) regular relationship with a man. Three quarters (77.5%) had engaged in anal intercourse with a man within 6 months. Half (49.4%) had tested HIV negative within the last 6 months, approximately one in 20 (4.6%) was living with diagnosed HIV, and a quarter (24.7%) had taken PrEP in the 6 months prior to the survey.

**Table 1:** Socio-demographic characteristics, sexual behaviours and HIV risk reduction among a national HIV behavioural surveillance sample of gay, bisexual and other men who have sex with men in NZ (n = 3235).

Characteristic*	N	%
Age		
16-29	1230	42.2
30-44	1054	36.2
45+	632	21.7
Ethnicity		
European only	2327	72.2
Any other ethnicity	898	27.8
Highest education		
Less than tertiary	1266	43.6
Tertiary or more	1638	56.4
Free time with gay men		
A lot	1086	33.6
Some, a little, none	2145	66.4
Sexual identity		
Gay identified only	2038	63.8
Bisexual, queer, other	1159	36.3
Current relationship status with a man		
Monogamous	740	23.4
Non-monogamous or unsure	873	27.6
No current regular male partner	1549	49.0
Anal intercourse with a man < 6 months		
Yes	2493	77.5
No	722	22.5
HIV test status		
Tested negative <6 months	1569	49.4
Tested negative more than 6 months ago	1067	33.6
Never tested for HIV	390	12.3
Diagnosed HIV positive	149	4.7
PrEP status		
Not taken PrEP < 6 months	2297	70.7
Taken PrEP < 6 months	801	24.7
Ineligible (HIV positive)	149	4.6

*May not total to N=3253 due to missing data

**Fig 1:**
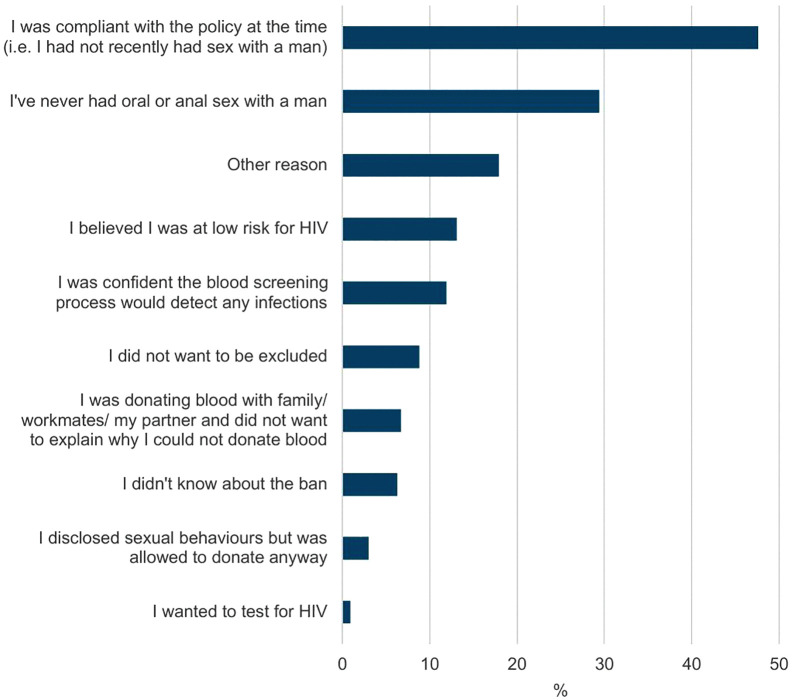
Reasons for having donated blood in the past among gay, bisexual and other men who have sex with men in NZ who had ever donated blood (n = 1393).

**Fig 2:**
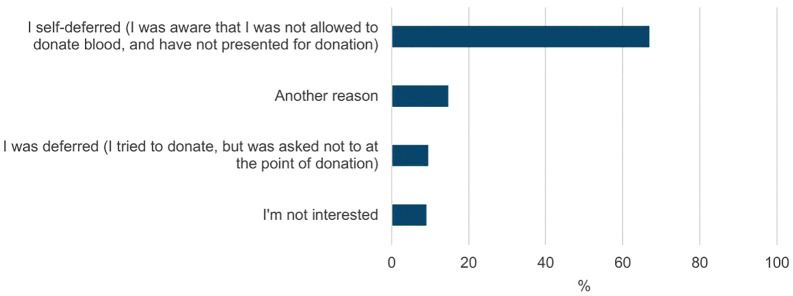
Reasons for not donating blood in the past among gay, bisexual and other men who have sex with men in NZ who had never donated blood (n = 1829).

Table 2 summarises key blood donation findings from the study. More than two in five (43.1%) of the 3235 participants had ever donated blood. Of those who had previously donated, the most common reasons were being compliant with the policy at the time, never having had oral or anal sex with a man, believing they were low risk for HIV, and being confident that screening processes would detect transfusion transmissible infections (Fig 1). “Other” reasons included “I was a teenager at school”, “It was before HIV arrived in NZ”, “I was straight then” and “My blood would still be helpful regardless of the fact I’m gay”. Among the majority who had never donated blood, most stated “I self-deferred”, whereas 9.5% stated they had presented to donate blood but were deferred (Fig 2). A substantial minority were not aware of the MSM deferral rule for donating blood (29.0%) or that people living with HIV faced a lifetime ban regardless of viral load status (20.9%), and most were unaware of the PrEP deferral criteria (72.7%) (Table 2). The majority of participants were interested in donating blood (82.0%), preferred an IDA-style deferral policy (86.1%), and stated they intended to donate blood in future, should they become eligible (80.6%).

**Table 2:** Blood donation history, interest, policy preferences and future intention to donate blood among a national HIV behavioural surveillance sample of gay, bisexual and other men who have sex with men in NZ (n = 3235).

Characteristic	N	%
Ever donated blood		
Yes	1393	43.1
No	1837	56.9
Awareness of MSM blanket deferral rule		
Aware	2250	71.0
Not aware	918	29.0
Awareness of PrEP deferral rule		
Aware	862	27.3
Not aware	2293	72.7
Awareness of HIV deferral rule		
Aware	2504	79.1
Not aware	661	20.9
Interested in donating blood		
Yes	2651	82.0
No	584	18.1
Future policy preference		
Previous blanket policy with no detailed behavioural questions	390	13.9
Individualised policy with more detailed behavioural questions	2420	86.1
Future intention to donate blood, if eligible		
Do not intend or neutral	579	19.4
Intend to donate	2410	80.6

MSM = men who have sex with men. PrEP = pre-exposure prophylaxis.

The deferral criteria for NZ’s previous 3-month MSM deferral policy were compared with those of the current IDA policies in the UK and Canada. Complete information relating to all deferral-relevant items was available from 2877 participants. Table 3 shows the deferral criteria domain, the specific options available from SPOTS (e.g., ever injected drugs) and self-reported prevalence of these characteristics among SPOTS participants, how these were “best matched” to each country’s deferral policy settings, and notes accompanying these decisions. Overall, we estimated that 13% of participants would have been be eligible to donate blood under NZ’s previous 3-month blanket-based rules for MSM. This would rise to 37% under the UK’s IDA rules, and 41% under Canada’s IDA rules (Table 3). The individual criterion triggering the largest deferral under NZ’s rules was having sex with a man in the previous 3 months (84.1% of participants). For the UK and Canadian IDA policies, anal intercourse with a recent casual partner deferred half (51.1%) of participants. For the UK, recent chemsex also deferred 18.2% of participants, a criterion not specified in the possible NZ or Canadian deferral policies.

**Table 3:** Potential eligibility of gay, bisexual and other men who have sex with men in NZ to donate blood based on 3-month MSM deferral policy, and hypothetical United Kingdom and Canada individualised donor policies (n = 2877).

				Participant deferred:		
Deferral item	Domain	Operationalisation of deferral criteria using available SPOTS questions	% deferred	In NZ’s 3-month MSM policy*	If NZ had adopted UK’s IDA policy**	In NZ’s new IDA policy based on Canada’s***
A	Age	Aged under 16 or over 70	1.1%	Y		
B	Age	Aged under 17 or over 66	3.1%		Y	
C	Age	Aged under 17	0.6%			Y
D	HIV	Living with diagnosed HIV	4.7%	Y	Y	Y
E	HIV	Current regular partner has HIV or last anal sex partner <3 months has HIV ^a^	2.4%	Y	Y	Y
F	HCV	Ever diagnosed with hepatitis C (even if cured)	1.5%	Y	Y	Y
G	STI	Ever diagnosed with syphilis (even if treated) ^b^	12.1%	Y	Y	Y
H	STI	Diagnosed with gonorrhoea <12 months ^c^	7.6%	Y		
I	PrEP	PrEP < 3 months ^d^	23.5%	Y	Y	Y
J	Sex with a man	Sex with a man < 3 months	84.1%	Y		
K	Anal sex	Anal intercourse with new (<3 months) current regular partner ^e^	3.8%		Y	Y
L	Anal sex	Anal intercourse with casual partner/s < 6 months ^f^	51.1%		Y	Y
M	IDU	Ever injected drugs	3.6%	Y	Y	Y
N	Chemsex	Any drugs for the purpose of sex < 6 months ^g^	18.2%		Y	
O	Sex work	Paying for sex < 3 months ^h^	1.7%	Y	Y	Y
P	Sex work	Been paid for sex < 3 months ^h^	2.0%	Y	Y	Y
	Deferred			87%	63%	59%
	Potentially eligible to donate blood			13%	37%	41%

NZ = New Zealand. UK = United Kingdom. IDA = individualised donor assessment. HCV = hepatitis C virus. STI = sexually transmissible infection. PrEP = HIV pre-exposure prophylaxis. IDU = injecting drug use.

* See notes below relevant to the NZ policy. ** See notes below relevant to the UK policy. *** See notes below relevant to the Canadian Blood Service policy (there is some regional variation in blood policy across Canada; these notes relate to the Canadian Blood Service).

a The Canadian criteria is deferral for people having sex with an HIV positive partners in the last 12 months.

^b^Although some policies have a 12 month deferral after syphilis treatment, the ongoing presence of antibodies to syphilis in blood will in practice result in that donated blood being discarded.

^c^The NZ criteria is deferral for 3 months after treatment for gonorrhoea.

^d^The Canadian criteria is deferral for 4 months after the last PrEP oral dose; in NZ and UK it is 3 months.

^e^The UK and Canadian criteria is deferral for 3 months following anal intercourse with a new partner.

^f^The UK and Canadian criteria is deferral for 3 months following anal intercourse with a new or multiple partners; SPOTS collected data on sex with casual partners over the last 6 months.

^g^The UK criteria is “drug use in the context of sex, except for Viagra and cannabis” in the last 3 months. The SPOTS question was “In the last 6 months, how often have you used drugs for the purpose of sex (e.g., chemsex, party’n’play, wired sex)?”.

^h^The Canadian criteria is deferral for 12 months after being paid for sex or paying for sex. The UK deferral is 3 months for being paid for sex in 3 months. The NZ deferral is for 3 months after being paid for sex.

Table 4 describes three groups based on these eligibility estimates (n = 2,873). Most (57%) participants were neither previously eligible nor likely to be in future. One in eight (13%) were eligible under NZ’s 3-month policy, and 30% were not eligible but would become so under NZ’s new IDA policy based on Canada’s. Newly eligible potential donors were more likely to be aged 30−44 (OR 1.8, 1.4–2.4) in comparison with currently eligible donors; they were also more likely to be tertiary educated (OR 1.4, 1.1–1.8), to spend a lot of their free time with gay men (OR 4.3, 3.2–5.9), and to identify as gay only (OR 2.5, 1.96–3.2). Those becoming eligible were less likely to identify as an ethnicity other than European-only (OR 0.7, 0.5–0.9). Table 4 also shows the behavioural profile of future-eligible MSM. Eighty-two per cent reported a regular relationship with a man at the time of survey (62.6% being monogamous and 19.0% being non-monogamous), 73.9% had engaged in anal intercourse with a man in the last 6 months, and 85.6% had received an HIV negative test. A small number (n = 13; 1.5%) reported using PrEP in the past 6 months, which includes participants taking PrEP 3–6 months ago, which is outside the NZ deferral window of 3 months.

**Table 4:** Socio-demographic characteristics and HIV-risk reduction behaviours of gay, bisexual and other men who have sex with men in NZ by the previous 3-month blanket deferral policy and future individual donor assessment policy eligibility status (n = 2873).

Characteristic	Not eligible previously nor in NZ’s new IDA policy from May 2026 (n = 1635)				Eligible to donate previously, in 3-month blanket MSM deferral policy (n = 379)			Eligible to donate in NZ’s new IDA policy from May 2026, but not previously* (n = 859)				Comparison of those eligible in NZ’s new IDA policy from 2026 versus those eligible previously
		**n**		**%**		**n**			**%**			**n**		**%**	**OR (95% CI)**
Age												
16-29	620		37.9		209		55.2	394		45.9		1
30-44	607		37.1		98		25.9	338		39.4		**1.83 (1.38-2.42)**
45+	408		25.0		72		19.0	127		14.8		0.94 (0.67-1.31)
Ethnicity												
European only	1147		70.7		261		69.4	662		77.3		1
Any other ethnicity	475		29.3		115		30.6	195		22.8		**0.67 (0.51-0.88)**
Highest education												
Less than tertiary	686		42.5		189		50.4	361		42.3		1
Tertiary or more	930		57.6		186		49.6	493		57.7		**1.39 (1.09-1.77)**
Free time with gay men												
A lot	565		34.6		56		14.9	368		43.1		**4.34 (3.17-5.94)**
Some, a little, none	1066		65.4		321		85.2	486		56.9		1
Sexual identity												
Gay identified only	1207		67.5		209		44.6	622		66.1		**2.52 (1.96-3.23)**
Bisexual, queer, other	580		32.5		260		55.4	319		33.9		1
Current relationship status with a man												
Monogamous	140		8.6		13		3.5	528		62.6		
Non-monogamous or unsure	629		38.8		9		2.4	160		19.0		
No current regular male partner	854		52.6		355		94.2	155		18.4		
Anal intercourse with a man < 6 months												
Yes	1531		94.3		77		20.3	626		73.9		
No	92		5.7		302		79.7	221		26.1		
HIV test status												
Tested negative <6 months	1113		68.9		73		19.8	236		28.2		
Tested negative more than 6 months ago	296		18.3		169		45.8	480		57.4		
Never tested for HIV	74		4.6		127		34.4	120		14.4		
Diagnosed HIV positive	133		8.2		0		0.0	0		0.0		
PrEP status												
Not taken PrEP < 6 months	801		49.0		374		98.9	844		98.5		
Taken PrEP < 6 months	701		42.9		4		1.1	13		1.5		
Ineligible (HIV positive)	133		8.1		0		0.0	0		0.0		

IDA = Individual donor assessment. MSM = men who have sex with men.

*Based on current Canada-style IDA deferral policy.

## Discussion

In this large and diverse community sample of MSM in NZ, we estimated that three times as many MSM will be eligible to donate blood as the country transitions from the previous 3-month blanket-based policy for MSM (13% were eligible) to a gender-neutral IDA policy in May 2026 based on the Canadian model (41% will now be eligible). Newly eligible MSM (approximately 30%) will differ demographically from previously eligible MSM, for example, they will be more gay community affiliated (in both identity and time spent with gay men), older, and have a higher education status. Many (43%) MSM in NZ have a history of donating blood, support shifting to an IDA policy, have an interest in donating blood, and report an intention to donate blood in future should they become eligible. However, over half (57%) of our participants would remain deferred from donating blood under NZ’s new IDA policy.

To our knowledge, our study is the first internationally to estimate the implications of a shift towards IDA in terms of both the volume and characteristics of newly eligible MSM donors. Our estimate that 41% of MSM are now eligible in NZ’s new IDA policy is similar to the findings from a nationally representative sample of 155 gay and bisexual men in Australia, that estimated 47.8% of MSM would be eligible to donate under a hypothetical future Australian IDA policy akin to those adopted internationally (in which MSM could donate if they had sex with another man in the previous 3 months and have had no new partner within the previous 12 months, and are not taking PrEP) [23]. However, it is not possible to directly compare the eligibility estimates, due to differences in study design, sampled populations and future IDA scenarios. A Canadian online cohort study estimated that 29.3% of 447 gay and bisexual men sampled would be eligible to donate blood based on reported sexual behaviours in the past 3 months [24]. However, this estimate did not include other potential deferral-triggering behaviours such as PrEP, drug use or STIs, as the purpose of the study was to compare eligibility under 12-month and 3-month MSM deferral policies. A USA in-person community study of 1593 MSM described deferral-relevant characteristics of participants under a USA IDA scenario mirroring that in the UK and Canada [25]. However, that study was designed to assess behaviours among MSM who were sexually active in the last 3 months, and excluded those who reported drug use, STIs and HIV, meaning our findings are not comparable. Given the narrow scope of existing research, more studies are needed to improve the range and reliability of findings.

We found that the criterion of having sex with a man (oral or anal intercourse) in the previous 3 months was responsible for the overwhelming majority (84.1%) of MSM deferrals under the previous NZ policy, representing missed opportunities to invite many MSM into donation efforts. We also found that if NZ had adopted the UK’s current policy at the time of the study, the chemsex criterion would defer 18.2% of MSM, resulting in 4% fewer MSM being eligible than under the Canada-style IDA policy that NZ finally adopted (the overall proportion deferred is lower, since chemsex behaviour overlaps with other deferral criteria). As long as the UK and Canadian policies are similarly safe for blood recipients, this suggests the Canadian policy will be more appealing to NZ in terms of improving the country’s blood supply.

Our demographic characterisation of newly eligible MSM donors (approximately 30% of MSM) can help the NZBS plan to welcome a new client base that has been previously excluded. Newly eligible donors will be more gay community-affiliated, older, and have a higher educational attainment than currently eligible MSM. As expected, many newly eligible donors will be in a regular relationship with a man, have engaged in anal intercourse in the last 6 months, and have a history of HIV testing. In other words, this represents a subgroup of MSM who display health-seeking behaviours and are at very low risk of having newly acquired undiagnosed HIV. Such insights can inform training for blood service staff, as well as the language, design, and images used in blood donation marketing. Their newfound eligibility to donate blood under an IDA policy is likely to be welcomed by gay communities in NZ, who have commonly pointed to this group as an example of MSM who should be able to donate blood based on their precautionary behaviour [26]. It also responds to critiques that blood donor policy often failed to account for the HIV risk-reduction practices of some MSM, which in turn led to feelings of distrust and frustration among gay communities against blood services and may have resulted in non-compliance amongst MSM presenting for donation [26,27].

Our findings, indicating that two out of five participants had previously donated blood, the majority of whom had done so prior to having sex with a man for the first time, are consistent with prior NZ research conducted during the 12-month deferral period [28]. Furthermore, over eighty per cent of all participants were interested in donating blood, supported an IDA policy even if it meant being asked more intrusive questions (such as having anal intercourse), and reported an intention to donate if they were eligible. Converting these positive views into donor presentation will be a crucial task for blood services. Previous NZ research has found that a sense of civic belonging among MSM was associated with donating blood, and active MSM donors were more common among MSM who were younger or students [27,29]. At the same time, some MSM participants in our study who had never donated blood reported that they were not interested, and qualitative research in NZ has noted some MSM who do not intend to donate blood had fears regarding blood or needles [11]. This underscores a need to avoid inadvertently stigmatising newly-eligible MSM who decline to donate. Some MSM may also prefer to retain their privacy rather than disclose more personal details. While emerging research is beginning to explore how to recruit MSM who have never donated before, future research must investigate the drivers of intentions to donate blood among the subgroup of MSM who are newly eligible in NZ, to identify factors the NZBS could efficiently target [30].

Despite the potential to attract more MSM blood donors, over half of MSM will still be ineligible under NZ’s new IDA policy. Among this group, 42.9% had taken PrEP in the last 6 months. Our study found low awareness among participants that PrEP use triggered deferral, implying that blood services should prepare information resources in light of evidence that PrEP use may mask breakthrough infections [31–34]. Likewise, 8.2% of MSM who will continue to be ineligible were living with diagnosed HIV, and in our study, not all participants were aware that having HIV triggered lifetime deferral. HIV community organisations have successfully socialised the concept of “U=U” (undetectable = untransmittable), reflecting the scientific consensus that someone with HIV who is diagnosed, treated and has an undetectable viral load cannot transmit HIV to their sexual partners [34,35]. However, U = U does not apply to donating blood, since the evidence does not rule out a risk of transmission to recipients [36]. Although MSM living with diagnosed HIV have expressed a strong desire not to place others at risk, some have welcomed the chance to help blood recipients if doing so could be safe [11]. To address these apparently conflicting messages, blood services should explain why donating blood is different to having sex regarding HIV transmission probabilities, even in the context of an undetectable HIV viral load.

Our study has several strengths. We used data from the largest and most diverse sample of MSM ever collected in NZ, increasing our statistical power to examine low prevalence characteristics. Participation was anonymous, reducing reporting biases regarding sensitive behaviours. We included granular questions about sexual behaviour and blood donation in the same instrument, many of which were tailored to previous and future deferral criteria (e.g., sex with a man in the last 3 months), enabling us to estimate the proportion of participants who would be deferred or eligible under different scenarios. The scenarios themselves (previous NZ, UK-style or Canada-style) reflected real-world options and were informed by advice from an international expert reference group with members from these countries. Our estimates of the potential eligible population can be triangulated with official statistics on gay and bisexual men in NZ to calculate the number of newly eligible donors, to help set donor recruitment targets and plan service delivery.

Our study also has limitations. The convenience-based recruitment strategy, non-random sampling, and lack of a national sampling frame for MSM, means we cannot know how generalisable our findings are to all MSM. However, our participants likely bias towards MSM who are more sexually active, overestimating the true proportion deferred and underestimating eligibility to donate among all gay and bisexual men. Blood donation was highlighted in our study promotion as one of four key themes (the others being HIV prevention, sexuality and sexual health care, and Māori MSM engagement), potentially disproportionately attracting MSM with an interest in this topic. We could not precisely match all deferral criteria in each country to our questionnaire items (e.g., the time period, or the exact behaviour); in these cases, proxies were used. The blood donation deferral items in SPOTS focused on behaviours relevant to MSM deferrals, and excluded general deferral-triggering behaviours or conditions (e.g., a recent tattoo, other health conditions, recent travel). This means our findings will underestimate the overall proportion deferred from donating blood for any reason within our sample. As our study focused on donor eligibility among MSM, we did not consider the potential impact on other donor populations of shifting towards IDA. For example, people’s willingness to answer more intrusive questions, or the potential reduction in eligibility among previously eligible heterosexual people [37].

## Conclusions

In conclusion, our study among MSM in NZ found that approximately four out of every 10 had previously donated blood, interest in donating blood was high, and the majority favoured a shift in policy towards individual donor assessment. At the time of writing, only 13% were eligible to donate under the country’s previous 3-month blanket MSM deferral policy, however this proportion could treble as NZ adopts a gender-neutral IDA policy modelled on Canada’s. Our findings on the increase in donor eligibility, coupled with the characteristics of potentially newly eligible MSM donors, support the case for change and can help blood services plan for IDA implementation.

## Abbreviations

HIV: Human immunodeficiency virus
IDA: Individual donor assessment
MSM: Men who have sex with men
NZ: New Zealand
SPOTS: Sex and Prevention of Transmission Study
TTI: Transfusion transmissible infection
